# Visualizing Similarity of Appearance by Arrangement of Cards

**DOI:** 10.3389/fpsyg.2016.00698

**Published:** 2016-05-13

**Authors:** Nao Nakatsuji, Hisayasu Ihara, Takeharu Seno, Hiroshi Ito

**Affiliations:** ^1^Graduate School of Design, Kyushu UniversityFukuoka, Japan; ^2^Faculty of Design, Kyushu UniversityFukuoka, Japan; ^3^Institute for Advanced Study, Kyushu UniversityFukuoka, Japan

**Keywords:** similarity, appearance, multidimensional scaling, typeface, visualization

## Abstract

This study proposes a novel method to extract the configuration of the psychological space by directly measuring subjects' similarity rating without computational work. Although multidimensional scaling (MDS) is well-known as a conventional method for extracting the psychological space, the method requires many pairwise evaluations. The times taken for evaluations increase in proportion to the square of the number of objects in MDS. The proposed method asks subjects to arrange cards on a poster sheet according to the degree of similarity of the objects. To compare the performance of the proposed method with the conventional one, we developed similarity maps of typefaces through the proposed method and through non-metric MDS. We calculated the trace correlation coefficient among all combinations of the configuration for both methods to evaluate the degree of similarity in the obtained configurations. The threshold value of trace correlation coefficient for statistically discriminating similar configuration was decided based on random data. The ratio of the trace correlation coefficient exceeding the threshold value was 62.0% so that the configurations of the typefaces obtained by the proposed method closely resembled those obtained by non-metric MDS. The required duration for the proposed method was approximately one third of the non-metric MDS's duration. In addition, all distances between objects in all the data for both methods were calculated. The frequency for the short distance in the proposed method was lower than that of the non-metric MDS so that a relatively small difference was likely to be emphasized among objects in the configuration by the proposed method. The card arrangement method we here propose, thus serves as a easier and time-saving tool to obtain psychological structures in the fields related to similarity of appearance.

## Introduction

Studies in psychology and cognitive science have attempted to structure the similarities among objects. Visualization of psychological structure has been intensively studied in the research field. The methods for visualization have been applied in practical-oriented study. For instance, Holleran (1992) visualized the relationship of similarity among 52 fonts as a map to extract common factors of fonts that people preferred. Chen (2009) also established a visualized map concerning 74 registered design patents for cars for the purpose of planning design strategies.

The geometrical approach has been employed to visualize the psychological structure. The geometric approach assumes that similarity or dissimilarity between the objects corresponds to the metric distances between the objects. Multidimensional scaling (MDS) is one of the geometric model approaches and has been commonly used for analyzing data, testing structural hypotheses, and exploring psychological structure in various field (e.g., Guttman, 1968; Borg and Groenen, 2005). Similarities between a pair of objects are transformed into a distance in a certain low-dimensional space and the objects are mapped into the space so as to satisfy each object's distance as best as possible. In general, the number of space dimensions is to be determined based on the fitting value, *Stress*, which expresses the errors between the similarity data and the distance (Kruskal, 1964a). The dimension can be selected so as to decrease the stress value.

This graphical display by MDS enables us to visually understand data structure even if there is no strong hypothesis that predicts patterns of data. For instance of analyzing data, Borg and Groenen (2005) provided a two-dimensional MDS representation regarding the correlations of crime rates over 50 U.S. states. Although it was difficult to understand the relationship behind the crime data without the MDS, it turned out that the crime data could be categorized by several items. That is, the horizontal axis and the vertical axis of the MDS representation can be interpreted as “violence vs. property” crimes and “hidden vs. street” crimes, respectively. The meaning of these axes is not obtained by MDS so we need to find rules of interpretation for describing MDS configurations by using additional knowledge.

Another object of MDS is testing structural hypotheses. For example, Levy (1983) categorized 18 types of attitudes toward political protest acts and confirmed that experimental results can reflect this organizational principle by using MDS. In this case, a three-dimensional MDS configuration was needed to explain the organizational principle appropriately.

MDS also enables us to discover the psychological structure that underlies similarity judgment. For instance, Wish (1971) collected similarity data among 12 nations from subjects and obtained the two-dimensional MDS configuration. The first axis of the two-dimensional map was interpreted as “pro-Western and pro-Communist” and the second axis as “economically developed and under-developed.”

When we explore the psychological structure of a subject by MDS, the direct method is often used for collecting similarity data. Asking subjects to evaluate a numerical value for each pair of objects, such as for the 9-point scale, is one of the ways to collect similarity data directly. Using numerical value as similarity data may cause possible problems in the psychological field, because the value of the subjects' psychological distance is not interval scale but ordinal scale. To resolve this problem, non-metric MDS has been introduced to use rank orders for similarity among objects to construct the object's configuration (e.g., Kruskal, 1964a,b). There are several procedures to collect order of similarity (Borg and Groenen, 2005). One is to ask subjects to sort cards from the highest similarity pair to the lowest one. Another method is asking subjects to classify the pairs of objects into two groups according to similarity. The pairs in each group are again classified into two groups in the same way. This procedure is repetitively performed until the subject thinks that it is no longer possible to find any differences in similarity among any pairs in the group. These methods are sometimes too time-consuming and demanding.

Although MDS is a useful tool as noted above, there are some problems in MDS techniques for exploring psychological structure within the realms of experimentation. Firstly, the subjects cannot assign meaning to MDS space. This makes it harder to understand one's psychological structure by translating the meaning of axes into a language, when there is no hypothesis or previous knowledge.

Secondly, when we try to obtain all combinations of similarity data between several objects, the subjects need to make a decision *n*(*n* − 1)∕2 times, where n is the number of the objects. If we take the subjects' workload into consideration, the number of objects should be limited to a relatively small number.

Thirdly, when the number of objects increases, it becomes difficult to find a solution for meeting the relationship between each similarity data and each distance on MDS space. In other words, the higher the number of objects is, the higher the stress value is (Spence and Ogilvie, 1973).

This study proposes a novel method to obtain the configuration of the psychological space of similarity data by experimental results without computational work. The proposed method asks subjects to arrange cards with stimuli on a poster sheet according to the degree of similarity between the cards, hereafter referred to as “card arrangement method.”

We expected that the configurations of MDS and the card arrangement method would be equivalent, because the configuration obtained by the card arrangement method should be reflected by the subjects' psychological space in the same way as by MDS. We also expected that this proposed method would enable us to obtain the configuration of psychological space in a shorter time than MDS, because subjects can arrange the cards while seeing, comparing, and moving all of the cards simultaneously.

In this study, we applied the card arrangement method and non-metric MDS, hereafter referred to as “nMDS,” to classify typefaces in order to clarify the common points and differences between the two configurations obtained by these methods and the advantage of the card arrangement method over MDS, including nMDS. We confirmed that each configuration of the card arrangement was approximately the same as that of nMDS. We found that the subjects exaggerated small differences between objects in the card arrangement method, which became a point of difference between the two methods. We also found that the experiment time of the card arrangement method was shorter than that of nMDS.

## Materials and methods

### Ethics statement

Our experiments were preapproved by the Ethics Committee of Kyushu University and informed written consent was obtained from each subject prior to testing.

### Subjects

Twenty volunteers participated in this experiment. They were divided into two groups comprising of ten subjects for each group, which were involved in two different experiments; namely, two conditions were employed according to a between-subjects design. The subjects were undergraduate students without expertise in the field of typeface design. They consisted of 12 males and 8 females, and their mean age was 21.05 (Min = 19, Max = 22, and *SD* = 0.86). All subjects were unaware of the exact purpose of this experiment and had normal or corrected to normal vision.

### Stimuli

We chose 10 typefaces from among the roman type, the sans-serif type, and the slab-serif type, which can be easily obtained because they are installed in Mac OS X by default. (i) Garamond, (ii) Baskerville, (iii) Bell MT, and (iv) Didot fall into the roman type; (v) Futura, (vi) Gill Sans, (vii) Helvetica, and (viii) Optima fall into the sans-serif type; and (ix) Rockwell and (x) Playbill fall into the slab-serif type.

**Figure 1:**
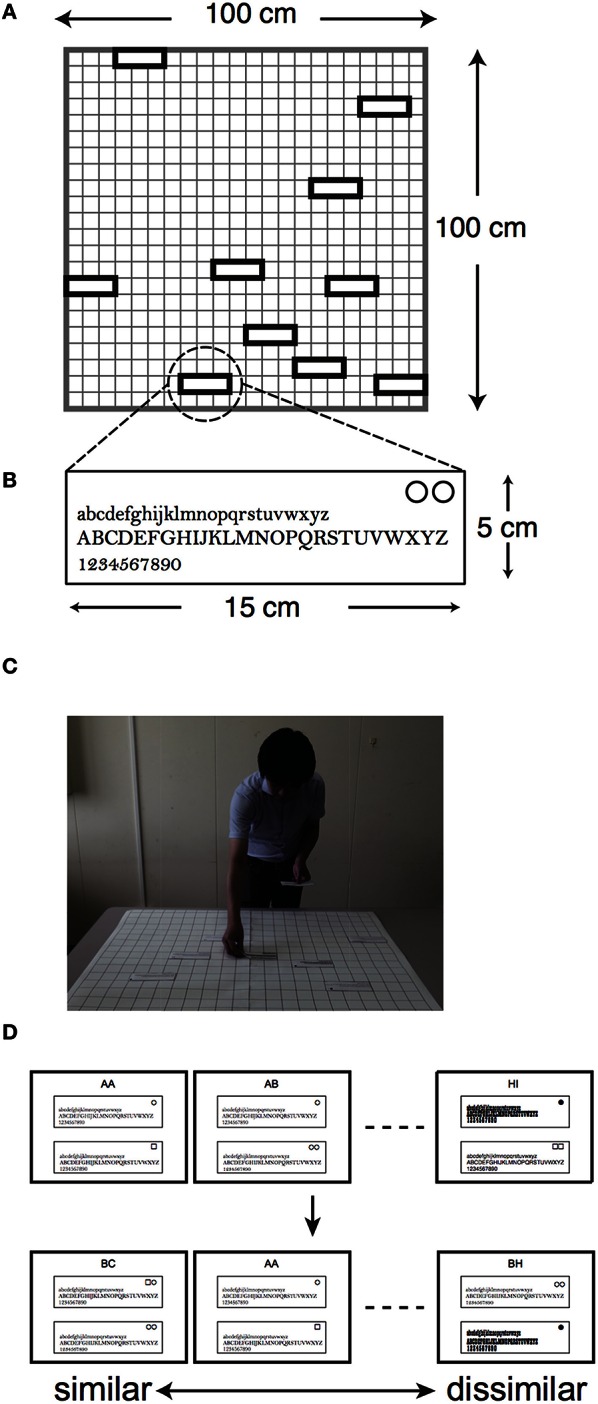
**Setup of the experiments**. **(A)** Schematic diagram of 10 cards arranged on the poster sheet in the card arrangement method. **(B)** Magnification of an example of stimuli for the card arrangement method. **(C)** Illustration of how a subject arranges the cards on the poster sheet. Each card contains alphabetical letters and Arabic numerals. There are identification marks in the upper-right corner of the cards. These cards are positioned by the subjects at certain distances depending on the degree of similarity regarding the appearances of the typefaces. **(D)** Examples of stimuli for nMDS. Each paper contains two kinds of typeface in the same way as those of the card arrangement method, and there are identification marks in the upper corner of the cards. The subjects rank the 45 papers according to how similar the two kinds of typefaces are while arranging all cards. The sorted order of the 45 papers is utilized for analysis of nMDS.

For the card arrangement method, we prepared 10 cards that each contained all lowercase and uppercase letters in alphabetical order (from “a” to “z” and from “A” to “Z”) and Arabic numerals (from “0” to “9”), as shown in Figure 1B. The cards were produced by PowerPoint (Microsoft). The colors of the cards and characters were white and black, respectively. The cards were rectangular in shape, measuring 5 cm in length by 15 cm in width, and each card weighed less than 1 g. The typeface size of the letters was 22 points, except for Playbill. Since height-to-width ratio of Playbill is much higher than the others, we assigned 40 points for Playbill to equalize the size of the typefaces in appearance. In order to have the subjects practice on the card arrangement method, six square-shaped cards were also prepared, measuring 15 cm in length by 15 cm in width. The six cards showed different shapes of a refrigerator; for example, one of the figures showed a two-door type refrigerator while another showed a three-door type.

For nMDS, we prepared 45 papers that each contained a different pair of typefaces. The papers were A-4 size and contained the same letters and numerals of the same size as those in the card arrangement method.

### Apparatus

For the card arrangement method, we prepared a white poster sheet of 100 cm square, with a 5 cm black grid onto which subjects were to place the cards (Figure 1A). We also prepared a digital camera (Power Shot SX 40, Canon) and a tripod stand to record the placement of the cards on the poster sheet. Furthermore, a desk of a certain size was prepared onto which the poster, digital camera, and tripod stand were to be placed. For nMDS, we prepared a desk onto which 45 cards could be placed. Both experiments were conducted in different rooms simultaneously, and each room had a luminance of around 400 lux.

### Procedure

For a practice on the card arrangement method, ten subjects were asked to place six cards with different refrigerators onto the sheet. The subjects were given instructions on how to adjust the position of those cards with similar refrigerators to a smaller distance. We wrote down the sentences “Being similar is arranged close-by” and “Being dissimilar is arranged farther away” on the white board to avoid confusion for the subjects. The subjects were also asked to use the entire space of the sheet and to match the corners of the cards to the corners of the grid. Even though the subjects assigned meaning to two axes of a two-dimensional space once, they were allowed to change the meaning of these two axes during the experiment. After the practice was completed, the ten subjects were asked to place the 10 cards with different typefaces in the same way as described above (Figures 1A–C). After arrangement of the cards, we took photos of the placements of the cards, checked the identification mark in the upper-right corner of the cards to identify them, recorded coordinates of the cards, calculated the Euclidean distances between each card as arranged on the sheet, and then considered the obtained distances as similarity data.

For nMDS, the other ten subjects ranked the 45 papers containing pairs of typefaces in order of similarity (Figure 1D). We collected a matrix of ordinal similarity data from the results. The nMDS configuration was obtained from the matrix based on Sammon's Non-Linear Mapping (Sammon, 1969).

Both experiments were carried out once for each of the subjects. Both the card placement in our proposed method and the evaluation of similarity in nMDS were continued until the subjects decided to finalize the process, so there was no time pressure in each condition. The subjects in both experiments were instructed to do the task as much as they like until they are fully satisfied.

The subjects evaluated their level of fatigue for the task and their satisfaction with the obtained configuration by providing ratings on two 5-point scales (1: not tired at all or not satisfied at all, 5: very tired or very satisfied). We also recorded the total time duration of the task.

### Quantification of similarity between configurations

We used a trace correlation coefficient (Hooper, 1959) to compare the configurations of the card arrangements with nMDS. Trace correlation coefficient takes a value between 0 and 1. The closer to 1 the value is, the more similar the pattern between two configurations is. Consider the matrix of configuration *X* = [*x*_1_, *x*_2_, ⋯*x*_*n*_], where column vector *x*_*i*_ is the Cartesian coordinate produced by [*i*]th stimuli. Let *X*^(*j*)^ be the configuration obtained by the [*j*]th subjects. The trace correlation coefficient between two of the configurations, *X*^(1)^ and *X*^(2)^, is defined as

$$\alpha \left(X^{\left(1\right)},X^{\left(2\right)}\right)=\sqrt{\frac{\rho_{1}^{2}+\rho_{2}^{2}}{2}},$$

where ρ_*i*_ is the [*i*]th canonical correlation coefficient between *X*^(1)^and *X*^(2)^. The value of trace correlation coefficient is invariant under the affine transformation of *X*. This property guarantees the same value of trace correlation coefficient can be obtained even if the configurations are rotated or parallel shifted. Thus we can compare two configurations through trace correlation coefficient regardless of their directions and shifts.

### Software

All statistical analyses and computations of the trace correlation coefficient were conducted using R version 3.2.1 (R Core Team, 2015) and R package “stats.”

## Results

### Examples of mapping

**Figure 2:**
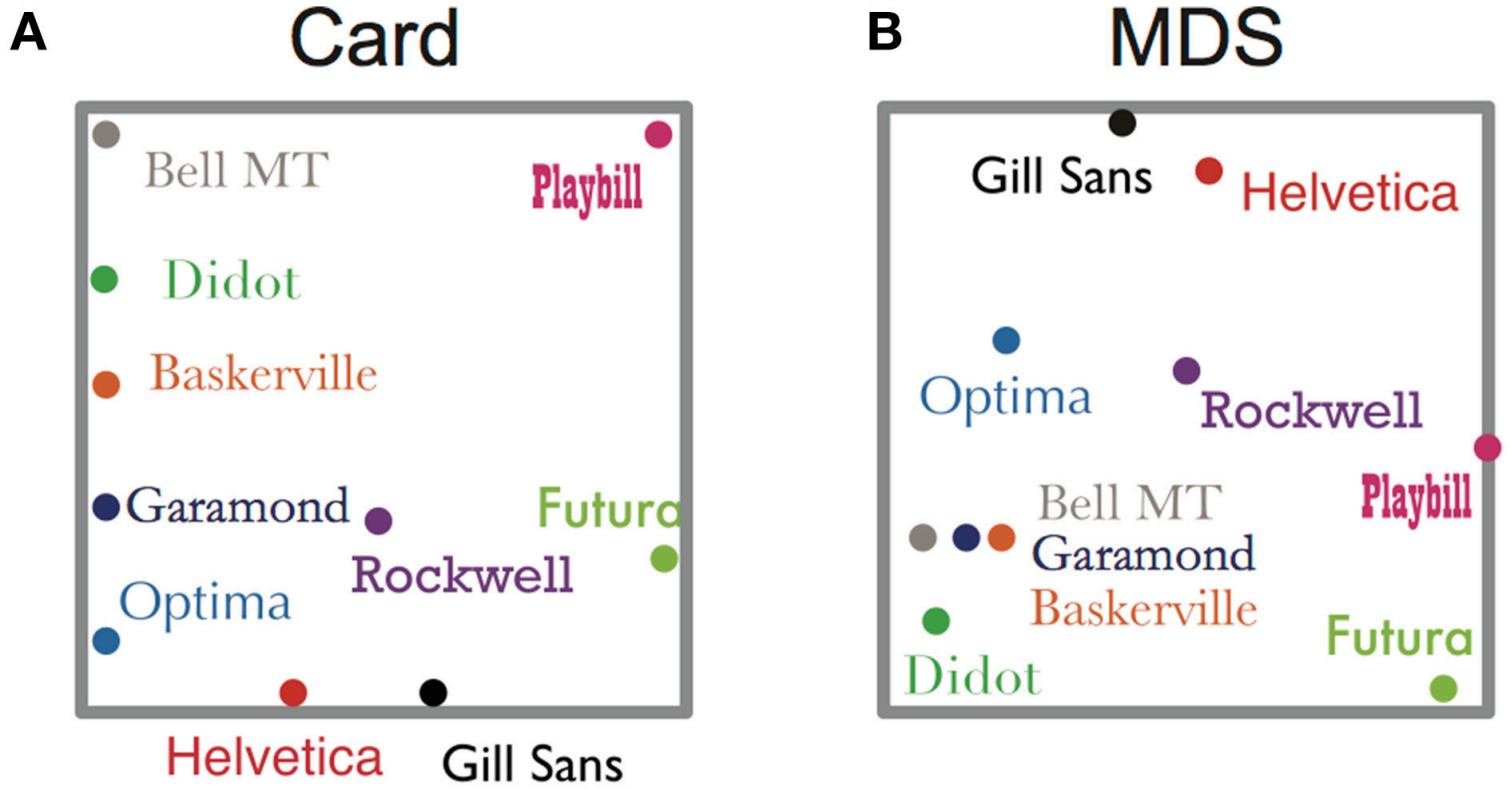
**Examples of configuration. (A)** Card arrangement method, **(B)** nMDS. The gray frame of **(A)** corresponds with a poster sheet onto which the cards are to be placed and the other gray frame of **(B)** shows MDS space after normalization. The same typefaces have the same color in both figures.

Figure 2 shows two examples of the configuration results of the card arrangement method and nMDS. As can be seen in Figure 2, Garamond, Baskerville, Bell MT, and Didot were placed at a short distance in both methods. It should be noted that the meaning of space in the two mapping methods is different. The axes shown in Figure 2B do not mean anything by the only principle of nMDS. They are supposed to be construed by additional knowledge such as serif type or non-serif type. In contrast, by using the card arrangement method, subjects can define the meaning of the axes. For instance, according to the subject whose result is shown in Figure 2A, the horizontal axis represents how bold or not bold the letters are, and the vertical axis represents how decorated or undecorated they are. As for all of the subjects, 90% of them responded that the first axis was how bold the letters are but the meaning of the second axis was not consistent among them.

### Difference level of configuration in space

In order to evaluate the similarity of the configuration patterns obtained by the two methods, we computed the trace correlation coefficient for each configuration pattern (see Material and Methods). We decided the threshold value of trace correlation coefficient for extracting similar pairs of configurations that is statistically significantly similar. The threshold value was defined based on random data as follows. The random configuration was generated by 10 pairs of random values following uniform distribution, *U*(0, 1). Thereafter, we calculated the trace correlation coefficient between a pair of random configurations 10^5^ times to obtain its distribution. The random trace correlation coefficient that showed a 0.05 level of significance corresponded to the trace correlation coefficient of 0.658, suggesting that 5% pairs of configuration by random data can show the trace correlation coefficient of more than 0.658. Thus, we chose 0.658 as the threshold value for similarity judgment.

**Figure 3:**
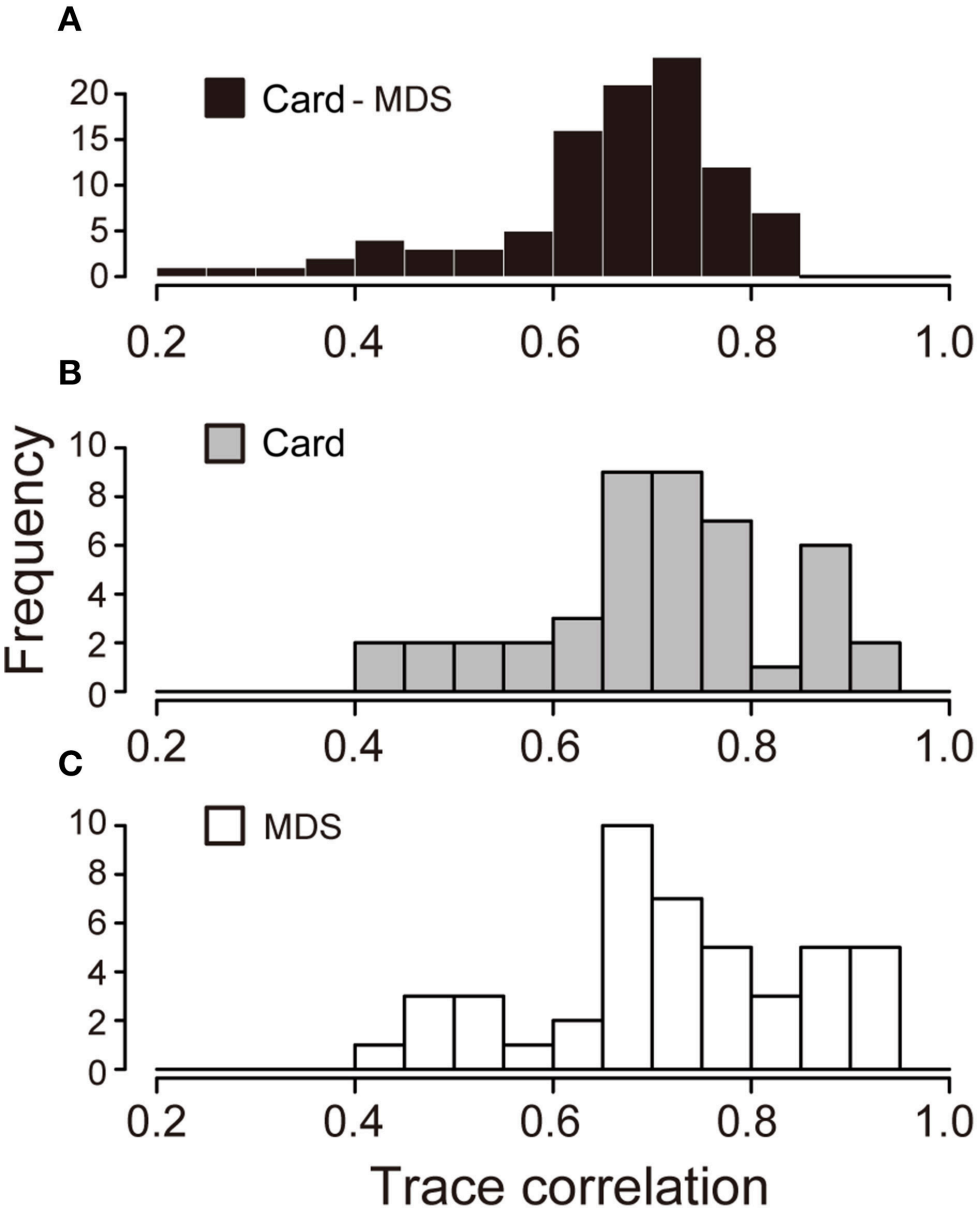
**Histogram of trace correlation coefficient among configurations. (A)** The histogram of trace correlation coefficient for all combination of configurations obtained by the card arrangement method and nMDS. **(B,C)** The histogram of trace correlation coefficient among all pairs of configurations by the card arrangement method and nMDS, respectively.

We calculated the trace correlation coefficient among all combinations of the configuration obtained from the card arrangement method and nMDS (Figure 3A). The ratio of the trace correlation coefficient exceeding the threshold was 62.0%. This result suggests that these methods can produce statistically significantly similar configurations.

We also examined variations of the subjects for each method. The trace correlation coefficient was calculated for all combinations of the configurations obtained by the proposed method and nMDS, respectively (Figures 3B,C). These distributions of the trace correlation coefficient have similar values for average and *SD*: Mean = 0.71, *SD* = 0.13 for the card arrangement method and Mean = 0.72, *SD* = 0.13 for nMDS, suggesting that both the methods have approximately the equivalent ability to express a psychological structure in similarity.

**Figure 4:**
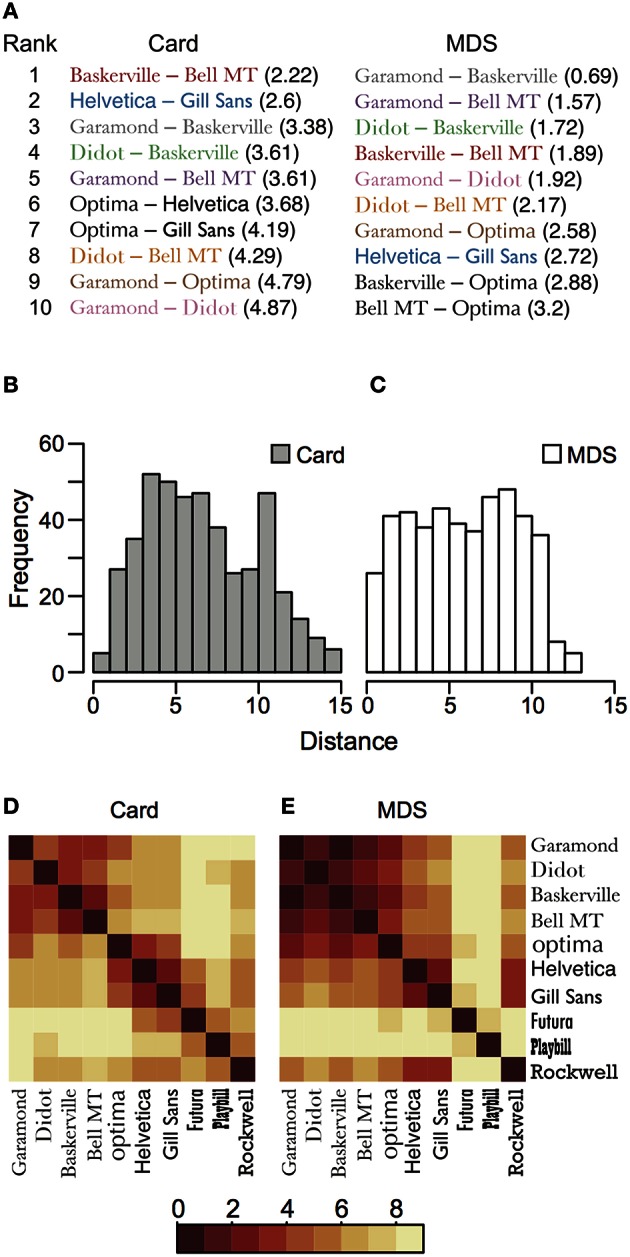
**Distance between the placed objects in card arrangement method and nMDS. (A)** The top 10 ranks of pairs with a small Euclidean distance in the configuration of both methods. The black-colored pairs are not shared in both methods, and the same-colored typeface pairs, other than black, represent common ones in both methods. **(B,C)** Histograms of distance between objects in the card arrangement method and nMDS, respectively. **(D,E)** Heat map of average of the distance between objects in the card arrangement method and nMDS, respectively. Darker color is assigned to the pair placed more closely.

Moreover, we examined the average of distance between the placed cards on the sheet and extracted the top 10 pairs with a small distance in each method (Figure 4A). Eight pairs among the top ten pairs are common in both methods. This also supports that there is a similarity between the configurations obtained from the card arrangement method and nMDS.

### Distances among objects are not close in configuration in the card arrangement method

Although we indicated the similarity in both methods, there are some differences in detailed point. For example, the configurations shown in Figure 2 had somewhat different features. Garamond, Didot, and Baskerville were located near each other in both the methods (Figures 2A,B), but these cards were arranged less closely in the card arrangement method (Figure 2A), compared with nMDS (Figure 2B). We assumed that subjects focused on the slight difference between objects and expressed their similarity judgment in a two-dimensional space, which may be the point of difference between the configurations of the two methods. In order to confirm this hypothesis, we calculated all distances between objects in all data (Figures 4B,C). We found the frequency of short distance to be lower in the card arrangement method compared with that in nMDS, which supports our hypothesis.

The average Euclidean distances between objects also support the hypothesis. In nMDS, Garamond, Didot, Baskerville, and Bell MT were placed close to each other (Figure 4E). However, in the card arrangement method, these typefaces were placed less closely (Figure 4D). Based on these results, we can conclude that card arrangement method tends to exaggerate the small difference between objects.

### Experiment time, level of fatigue, and level of satisfaction

**Figure 5:**
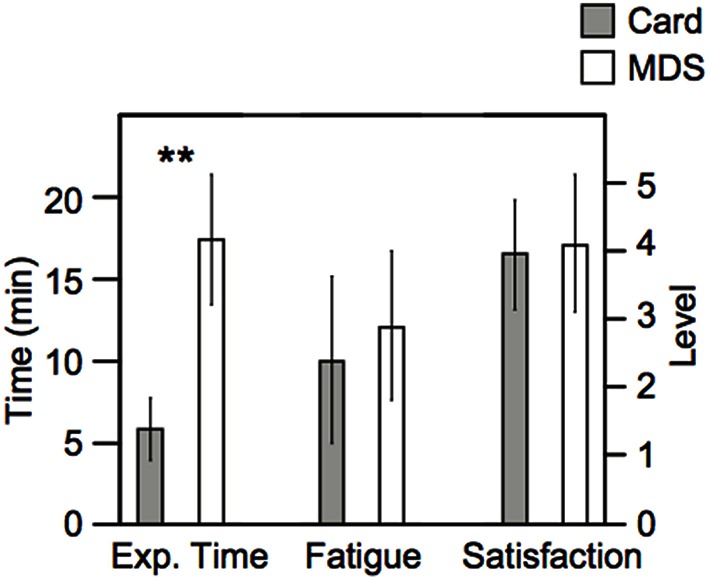
**Total duration of experiment time, the results of subjective fatigue, and satisfaction**. The left axis represents the scale for experiment time. The right axis represents the level of fatigue and satisfaction. The error bar indicates the standard deviation of each result. ^**^ Indicates statistical significance determined by *t*-test (*p* < 0.01).

The required duration of the card arrangement method was drastically shorter than that of nMDS, as we expected (Figure 5). The duration of the card arrangement method was approximately one third of nMDS's duration. Regarding the level of fatigue and satisfaction, no significant difference between the two methods was observed.

## Discussion

This study attempted to clarify common and different points between the configurations obtained by the card arrangement method and nMDS and the advantage of the proposed method. We found that there is a correlation between the configurations obtained from the proposed method and those obtained from nMDS. This result demonstrates the fact that the proposed method can be used instead of nMDS to obtain psychological structures. The required duration for the proposed method is considerably shorter than that required for nMDS, which indicates that the proposed method is a useful tool to save time. In particular, the proposed method can be utilized effectively in the psychological field, because evaluation of similarity must be obtained from subjects directly and their workload should be reduced. While this study asked subjects to sort papers from the highest similarity pair to the lowest one and collected ordinal similarity data for nMDS, the required duration for the proposed method would be shorter than that of the other collecting method for ordinal similarity, such as forming groups corresponding to the degree of similarity. Moreover, the proposed method is highly simple and easy since it requires only cards and poster sheets while nMDS requires relatively complicated data analysis.

We understand that this proposed method could be applied to existing research related to similarity of appearance. For example, Holleran (1992) asked 50 subjects to rate the similarity between 52 typefaces and tried to map the subjects' psychological structure of similarity into two dimensions. If we apply the card arrangement method to these stimuli, we could obtain similarity data and the configuration of the stimuli in a markedly shorter time. In addition, Holleran (1992) did not specify the meaning of horizontal axis and vertical axis, but we could accomplish this by using the card arrangement method, because we could directly obtain the axes' meaning from the subjects.

The Management and Law fields also have a need for similarity of appearance analyses. Enterprises file applications and obtain design patent rights to prevent their appearance of products from imitation. They develop a design patent map from publication of the design patent not only to manage their own designs already filed but also to ensure positioning between their product design and their competitor's design. In the design patent map, each design should be arranged based on the degree of similarity among them. For example, Chen (2009) collected similarity data regarding 74 registered design patents for cars from industrial designers, established a design map by using MDS, and drafted the design map for the purpose of planning design strategies.

In addition, once the infringement lawsuit is filed, similarity of the registered design is becoming a material matter, because there is a possibility that they have to stop selling their products and pay damage. In the practice of major countries regarding design patent infringement, if the registered design and an accused design are the same or similar and give a common impression, it is judged that the accused design violates the rights of the registered design (e.g., Article 23 of the Japan Design Act^1^; Egyptian Goddess v. Swisa, 2008^2^; Article 10 of Council Regulation (EC) No. 6/2002 of 12 December 2001 on Community Designs^3^). Also, when the accused design is compared to the registered design, the prior art should be taken into account. For example, 10 prior designs were submitted to decide the essential part of the registered design in a certain Japanese infringement case (Hei-12 (Wa) 2240, 2001^4^). If MDS were to be applied to such a case, the experiment would take much time due to the large number of stimuli. The method proposed in this study could be applied to such a situation to save experiment time. Moreover, there are many cases where the registered design and the accused design appear very similar at first glance. In such a case, we can make good use of the proposed method, because subjects can focus on slight differences when they make similarity judgments according to this method.

The subjects can watch all the cards and evaluate similarity among them simultaneously in the proposed method, so that the results of the proposed method can be affected by context. It is known that the similarity of objects A and B are influenced by object C (Tversky, 1977). In the proposed method, subjects can see and compare all the objects while arranging cards so that all the objects can be considered when evaluating the similarity between objects A and B. On the other hand, regarding the pairwise comparison, subjects can see only A and B and evaluate the similarity of A and B so that C would not be taken into account. Thus, if we expect the results to be affected by the context effect, the proposed method should be applied. Otherwise, the pairwise comparison would be better.

The proposed method allows the subject to set the axis of the configuration pattern. In this case, the meaning of the axis can be revealed through the speech of the subjects. In contrast, there is a case where the subjects do not recognize the axis even after finishing the task. In this instance, someone other than the subjects can give meaning to the axis from a two-dimensional configuration. MDS enables us to find a principle dimension even if the subject does not note the meaning of the dimension, which is one of MDS' benefits. In that sense, the merit of MDS is shared with the proposed method.

The proposed method is confined to a two-dimensional space. The two-dimensional space is the easiest dimension to be interpreted and applied to actual issues. Although compressing psychological structure into a two-dimensional space can reduce the amount of information that the original psychological structure preserves, one can estimate the degree of reflection for cognitive space by asking the subjects about the “degree of satisfaction of configuration.” The extension of our model to a three-dimensional space may be possible, when the two-dimensional space is not large enough to reflect one's cognitive structure. The development of a software that allows us to place objects in a three-dimensional space and validating of the effectiveness of such a space may be explored in future works.

The proposed method is effective for visual stimuli because subjects can watch all cards and evaluate them simultaneously. Moreover, this method may be applied to conceptual stimuli, such as similarity between nations. We expect that this card arrangement method has versatility and that it will be possible to apply it to various areas. The application to other fields should be explored in future studies.

## Author contributions

NN, HIhara, and HIto conceived and designed the study; NN and HIto performed the experiments; NN and HIto analyzed the data; and NN, TS, and HIto wrote the paper.

### Conflict of interest statement

The authors declare that the research was conducted in the absence of any commercial or financial relationships that could be construed as a potential conflict of interest.

## Footnotes

^1^http://www.japaneselawtranslation.go.jp/law/detail/?id=44&vm=04&re=01.

^2^http://www.cafc.uscourts.gov/content/egyptian-goddess-v-swisa-inc.

^3^http://eur-lex.europa.eu/legal-content/EN/TXT/HTML/?uri=CELEX:32002R0006&rid=4.

^4^http://www.courts.go.jp/app/hanrei_jp/detail7?id=12556.
